# Long-Term Oxidation Susceptibility in Ambient Air of the Semiconductor Kesterite Cu_2_ZnSnS_4_ Nanopowders Made by Mechanochemical Synthesis Method

**DOI:** 10.3390/ma16186160

**Published:** 2023-09-11

**Authors:** Katarzyna Lejda, Magdalena Ziąbka, Zbigniew Olejniczak, Jerzy Franciszek Janik

**Affiliations:** 1 Faculty of Energy and Fuels, AGH University, al. Mickiewicza 30, 30-059 Krakow, Poland; 2 Faculty of Materials Science and Ceramics, AGH University, al. Mickiewicza 30, 30-059 Krakow, Poland; 3 Institute of Nuclear Physics, Polish Academy of Sciences, ul. Radzikowskiego 152, 31-342 Krakow, Poland

**Keywords:** kesterite semiconductor, energy band gap, mechanochemical synthesis, nanopowders, oxidation in air, kesterite oxidation products

## Abstract

The often overlooked and annoying aspects of the propensity of no-oxygen semiconductor kesterite, Cu_2_ZnSnS_4_, to oxidation during manipulation and storage in ambient air prompted the study on the prolonged exposure of kesterite nanopowders to air. Three precursor systems were used to make a large pool of the cubic and tetragonal polytypes of kesterite via a convenient mechanochemical synthesis route. The systems included the starting mixtures of (i) constituent elements (2Cu + Zn + Sn + 4S), (ii) selected metal sulfides and sulfur (Cu_2_S + ZnS + SnS + S), and (iii) in situ made copper alloys (from the high-energy ball milling of the metals 2Cu + Zn + Sn) and sulfur. All raw products were shown to be cubic kesterite nanopowders with defunct semiconductor properties. These nanopowders were converted to the tetragonal kesterite semiconductor by annealing at 500 °C under argon. All materials were exposed to the ambient air for 1, 3, and 6 months and were suitably analyzed after each of the stages. The characterization methods included powder XRD, FT-IR/UV-Vis/Raman/NMR spectroscopies, SEM, the determination of BET/BJH specific surface area and helium density (d_He_), and direct oxygen and hydrogen-content analyses. The results confirmed the progressive, relatively fast, and pronounced oxidation of all kesterite nanopowders towards, mainly, hydrated copper(II) and zinc(II) sulfates, and tin(IV) oxide. The time-related oxidation changes were reflected in the lowering of the energy band gap E_g_ of the remaining tetragonal kesterite component.

## 1. Introduction

The quaternary sulfide, Cu_2_ZnSnS_4_, and its selenized derivative, Cu_2_ZnSn(S,Se)_4_, customarily called kesterite, have been in recent years considered for use in the active layers of photovoltaic (PV) cells as alternative semiconductors to the currently dominant silicon (Si) varieties [1,2,3,4]. Kesterite is characterized by the suitable energy band gap for solar light conversion in the range 1.0–1.5 eV, a large absorption coefficient (>10^4^ cm^−1^), and its composition of readily available non-toxic elements that are environmentally friendly both while in use and after disposal. The compound is made primarily in the form of polycrystalline bulk powders or as thin films, the latter often accomplished by substrate powder processing to make/mimic the kesterite PV layered devices [5,6,7]. Occasionally, bulk batches of kesterite can be prepared with crystallite sizes large enough to be studied by single-crystal-specific techniques [8]. One of the convenient preparation methods in the solid state is a high-energy ball milling of suitable precursors, which yields the nanocrystalline kesterite powders via mechanochemical interactions [9,10]. In this area, in recent years, we have mastered the preparation of Cu_2_ZnSnS_4_ nanopowders from a few precursor systems [11,12,13] as well as showed the feasibility of the nanopowders under high-pressure (7.7 GPa) and high-temperature (500 °C) for sintering towards mechanically robust kesterite nanoceramics [14].

Many practical aspects of kesterite synthesis, storage, and utilization are a function of the quaternary sulfide’s susceptibility to water-vapor-assisted oxidation in an ambient air atmosphere. These often overlooked circumstances may have both negative and positive repercussions in the synthesis and manipulation of kesterite, as revealed by some relevant topical reports [15,16,17,18,19,20,21,22,23]. From these reports, it is evident that, usually, the major oxidation products that are formed rather slowly under close to ambient experimental conditions are the hydrates of copper and zinc sulfates, while no unequivocal data are presented regarding the tin component. On the other hand, at higher temperatures of the order of 300–400 °C, thin films of kesterite are shown to oxidize in air with the formation of crystalline SnO_2_ and ZnS + Cu_2_S phases, whereas at 650 °C, in addition to SnO_2_, ZnO and CuSO_4_ are detected [24]. Also, in the earlier mentioned study on kesterite sintering (7.7 GPa, 500 °C), the formation of some crystalline SnO_2_ was identified in the nanoceramics, apparently, due to a presence of adventitious oxygen in the nanopowder substrates and/or some kesterite nanopowder oxidation past the synthesis stage.

The oxidation, both adventitious and intentional, of the individual binary sulfides of copper, zinc, and tin was extensively investigated, mostly, addressing many practical aspects of the respective metal sulfide ores utilization. In this regard, the oxidation of Cu_2_S in dry air at temperatures of up to 850 °C confirmed the stepwise formation of CuO via the intermediate Cu_2_O, while no transient copper sulfate, CuSO_4_, was found [25]. However, other studies on the oxidation (air, nitrogen/oxygen, oxygen) of powdered Cu_2_S and CuS at temperatures starting as low as 250 °C confirmed a temperature-dependent course of oxidation reactions, with CuO being eventually formed at the highest temperatures in the range of 700–850 °C, whereas CuSO_4_ and CuO•CuSO_4_ were detected at the temperatures as high as 450–700 °C [26]. The surface oxidation of chalcocite ore, Cu_2_S, at ambient conditions upon exposure to air for seven days or in air-saturated aqueous slurries resulted in the former case, i.e., in the detection of Cu_2_O and sulfate groups [27] that were also detected by XPS measurements in the CuS and Cu_2_S powders made by high-energy ball milling [28]. The thermogravimetric study of chalcocite under an atmosphere of dry air or air with added moisture confirmed the formation of copper sulfate starting at 340 °C, while the humid air favored a relatively faster oxidation reactions, especially, for the samples ground to the smallest particle sizes [29]. These studies are consistent with the phase diagram in the system Cu-S-O, which supports the notion that the temperature-driven oxidation of copper sulfides initially results in the formation of copper sulfates and/or oxysulfates that may decompose to copper oxide, CuO, sometimes through the intermediate Cu_2_O at increased temperatures [30].

Following the traits of many metal sulfide ores, zinc sulfide, ZnS, was reported as early as in 1910 to be oxidized in the ambient air towards zinc sulfate [31]. In a study on oxidation (invloving close to ambient conditions, controlled humidity, and a period of time up to 5 weeks) of the sulfide ores of lead, zinc, and copper, the exclusive formation of the sulfates of lead and zinc, often in hydrated forms, was confirmed, whereas in the case of chalcocite ore (Cu_2_S) the inferred oxidation products were CuO, CuS, and Cu(OH)_2_ [32]. In another study on the high temperature oxidation of ZnS in the atmosphere of pure oxygen or oxygen supplemented with water vapor, zinc sulfite [SO_3_]^−2^ and sulfate [SO_4_]^−2^ products were identified up to the temperature decomposition range of the latter, i.e., exceeding 600 °C [33]. At still higher temperatures in the 840–1000 °C range, the zinc sulfide in the sphalerite ore was oxidized to ZnO [34]. It is obvious that the oxidation products of zinc sulfide in the air depend greatly on the temperature and the presence of water vapor while the hydrated zinc sulfate is preferentially formed at ambient.

The susceptibility of tin sulfides [35], e.g., Sn(II)S, Sn(II)Sn(IV)S_3_, and Sn(IV)S_2_ to ambient oxidation follows a different pattern compared to the sulfides of copper and zinc, and it is specific in that various tin compounds are chemically amphoteric and many of them, including salts, are subject to hydrolysis in the presence of water. Although, the single crystal structures of some tin sulfates such as tetravalent Sn(SO_4_)_2_ and heterovalent Sn_2_(SO_4_)_3_ have been recently resolved [36], the chemistry of the salts with respect to water under common laboratory conditions (wide range of pH, excess of water/diluted solutions, ambient temperatures) leads rather to their hydrolysis and precipitation of insoluble, highly amorphous hydrated tin(IV) oxide as exemplified for {Sn(SO_4_)_2_ + yH_2_O → ↓SnO_2_•xH_2_O + ↑2SO_2_ + (y−x)H_2_O} [37]. However, in the case of the hydrolysis of the divalent tin(II) sulfate SnSO_4_ under selected conditions, a complex hydrated tritin(II)dihydroxyoxosulfate Sn_3_O(OH)_2_SO_4_ was formed [38]. In this regard, the crystalline Sn(SO_4_)_2_ and Sn_2_(SO_4_)_3_ were prepared and skillfully isolated from the reactions of tin oxide SnO with the concentrated sulfuric acid, oleum (65% SO_3_), and B(OH)_3_ to avoid the hydrolysis. The somewhat ambiguous water insoluble solid SnO_2_•xH_2_O, often called even nowadays the stannic/metastannic acid, has the *x*-value occasionally quoted as 1 to correspond with the formal stannic acid H_2_SnSO_3_, while the data from the scarce and often very mature literature references point out rather to the various hydrated forms of tin(IV) oxide SnO_2_•xH_2_O [39,40]. The important aspect of the hydrolysis-promoted formation of the colloidal hydrated tin(IV) oxide is its amorphous nature which renders the application of powder XRD of limited use for its detection. It should also be noted that the oxidation of tin sulfides, both SnS and SnS_2_, in the air at sufficiently high temperatures in the range 400–600 °C, results in the formation of SnO_2_ [41]. 

Given the significance of kesterite oxidation in the atmosphere of air, herein, a study is presented on an extended in time exposition to ambient air of the semiconductor kesterite Cu_2_ZnSnS_4_ nanopowders. The nanopowders are prepared by high-energy ball milling that promotes the mechanochemically assisted synthesis of kesterite. Three different precursor systems are applied to yield a large pool of kesterite products both raw (cubic polytype tentatively called prekesterite) and annealed at 500 °C under argon (tetragonal polytype called kesterite). The important structure and spectroscopic material properties are followed up to 6 months of exposure to address a progress of oxidation processes on this time scale.

## 2. Experimental

### 2.1. Preparation of Kesterite Nanopowder Materials 

Three precursor systems were used for mechanochemical synthesis of the kesterite materials via “wet” high energy ball milling of substrates in xylene (Pulverisette 7, Fritsch, Idar-Oberstein, Germany) as described by us in the earlier reports. The first system was made of the constituent elements (CE) in the stoichiometric proportion, i.e., copper Cu, zinc Zn, tin Sn, and sulfur S with 2 at% excess of S that were milled for 16 h at 1000 rpm [11]. Upon overnight xylene evaporation, the resulting solid prekesterite was sampled for characterization and used in the thermal annealing under argon at 500 °C for 6 h to yield a black kesterite nanopowder. In the second system, a stoichiometric mixture of the metal sulfides (MS), i.e., copper(I) sulfide Cu_2_S, zinc(II) sulfide ZnS, tin(II) sulfide SnS, and sulfur S with 2 at% excess of S was milled for 20 h at 900 rpm [12]. After xylene evaporation, the resulting prekesterite nanopowder was characterized and, then, used in the thermal treatment at 500 °C under argon for 6 h to produce a black powder of kesterite. In the third system, the in situ made copper alloys (CA) from high energy ball milling of the metal powders {2Cu + Zn + Sn} for 10 h at 900 rpm were further milled with a 2 at% excess of sulfur vs. stoichiometry for 4 h at 900 rpm to yield a prekesterite nanopowder [13]. The latter material was annealed under argon at 500 °C for 6 h to result in the kesterite nanopowder. After sampling for characterization (samples labeled “fresh” or “freshly made”) and further processing, the nanopowders were stored in the desiccator until all materials from the three precursor systems were prepared and characterized. For the oxidation study, approximately 5 g of each powder was evenly spread with a thickness of ca. 1 mm on an individual glass dish and all six dishes were placed next to each other under the fume hood. The nanopowders were sampled for characterization after 1, 3, and 6 months. At the longest exposure time, some sample color lightning was observed. Similar experiment was performed for the starting metal sulfide powders used in the MS precursor system. Each of the powders of Cu2S, ZnS, and SnS was placed on an individual glass dish, spread as a thin layer, and exposed to ambient air together with the kesterite samples. In this case, the characterization was done for the batches of the sulfides from freshly opened containers and for the powders after one month of exposure to air.

### 2.2. Sample Labeling

The samples were named upon their original precursor system and the stage of nanopowder processing with the latter referring to either (i) freshly made powders with no intentional exposure to air—raw nanopowders of (cubic) prekesterite and annealed at 500 °C nanopowders of (tetragonal) kesterite or (ii) time of air exposure—nanopowders after 1, 3, 6-month exposure to ambient air. The CE label was used for the system made of the constituent elements, MS for the system of metal sulfides, and CA for the system made via intermediate copper alloy formation.

### 2.3. Characterization

Powder XRD determinations were conducted for all nanopowders on Empyrean PANalytical (Malvern, UK), Cu Kα source, 2Θ = 10–110°, and average crystallite sizes were estimated from Scherrer’s equation. An ultra-high-resolution analytical FIB-SEM Scios 2 (Thermo Fisher Scientific, Waltham, MA, USA) was used for morphology observation. Powders were placed on a conductive carbon tape and coated with a 10 nm carbon layer (EM ACE600 sputter coater, Leica Microsystems, Wetzlar, Germany) then observed at an accelerated voltage of 10kV, under high vacuum, using in-column detector (T2) and opti-plane mode. FT-IR spectroscopy (Nicolet 380, Thermo Electron Corp., Waltham, MA, USA) was carried out on KBr pellets containing about 1 mg of samples. Raman spectroscopy was performed on a WITec Alpha 300M+ spectrometer (WITec, Ulm, Germany) equipped with Zeiss optics (50×) and a 488 nm diode laser. Four accumulations of 30 s scans were collected at each point. Baseline subtraction was accomplished with WITec’s software (ProjectFive Plus, WITec, Ulm, Germany). Deconvolution of spectra was done using a mixed Gaussian-Lorentzian curve fitting. UV-vis measurements were carried out with a Perkin-Elmer spectrophotometer Lambda 35 equipped with a 50 mm integrating sphere for powder samples. Solid-state MAS NMR spectra were recorded on the APOLLO console (Tecmag) at the magnetic field of 7.05 T with the Bruker HP-WB high speed MAS probe equipped with the 4mm zirconia rotor and KEL-F cap, which was used to spin the sample. The ^65^Cu NMR spectra were determined at 85.11 MHz with the spinning speed of 4 kHz. The frequency scale in ppm was referenced to the ^65^Cu resonance of CuCl. The ^119^Sn NMR spectra were measured at 111.68 MHz with the spinning speed of 4 kHz. The frequency scale in ppm was secondary-referenced to the central transition of SnS spectrum located at −299 ppm. BET (Brunauer-Emmett-Teller)/BJH (Barrett–Joyner–Halenda) specific surface areas were determined from low-temperature nitrogen adsorption isotherms on Micromeritics Gemini 2380 (Norcross, Ga, USA). Helium densities were obtained with a Micromeritics AccuPyc 1340 pycnometer (Norcross, GA, USA). The d_He_ values for the samples were rounded up to the nearest 0.01 g/cm^3^ to show them with accuracy exceeding one standard deviation value in each case. The oxygen and hydrogen contents were directly determined with the ONH836 elemental analyzer (Leco Corporation, St. Joseph, MI, USA) using 0.01–0.02 g of a sample.

## 3. Results and Discussion

The application of three precursor systems in the mechanochemically assisted synthesis of kesterite Cu_2_ZnSnS_4_ nanopowders is aimed at providing a large pool of chemically the same but synthetically slightly non-uniform materials for the oxidation study. The materials are characteristic of the same preparation method while being “imprinted” with different initial oxygen contents of the substrates and their oxidation susceptibility as well as having varying structure (extent of lattice order/disorder, variation of lattice parameters) and morphological features (ranges of average crystallite size, specific surface area, and helium density). The mechanochemical synthesis is specific in that the raw product isolated after the high energy ball milling is a nanopowder of the kesterite’s cubic polytype, called tentatively by us prekesterite, that does not show any semiconductor properties due to intrinsic d_0_ magnetism [11,12]. The thermal annealing, usually at 500 °C under neutral gas atmosphere, is subsequently applied to convert this material to the tetragonal kesterite semiconductor. The pool of samples is therefore made of the nanopowders of three prekesterites and three kesterites which were prepared as described in Experimental. Of note is the applied standard way of drying the raw xylene slurry after milling, which was done by an overnight (ca. 12 h) evaporation of xylene from an opened to air grinding bowl placed under the fume hood. This afforded a free flowing blackish powder that was then stored in the desiccator for further characterization and processing.

In our recent paper on the oxygen-related aspects of the high-pressure and high-temperature sintering of kesterite nanopowders, we confirmed noticeable oxidation phenomena occurring in the synthesis as evidenced by the presence of crystalline tin(IV) oxide SnO_2_ in the kesterite nanoceramics [14]. Additionally, oxygen-bearing copper and zinc sulfates were seen by FT-IR in the substrate nanopowders, which was supported by the evolution of sulfur(IV) oxide SO_2_ in the TGA-DTA/MS experiments and by direct oxygen content determinations. The extent and rate of oxidation could only be roughly estimated since, despite considerable attempts to follow standardized manipulation/determination procedures, quite widely scattered characterization data were obtained for the examined pool of materials. In the current approach, we target the major oxidation events for the pool of kesterite nanopowders exposed together to the same ambient air conditions for the period of time up to 6 months. The nanopowders are characterized as freshly made and, later, after 1, 3, and 6 months of being exposed to air. Such a long period of time proved to be sufficient for providing the unequivocal evidence about the nature and rate of kesterite nanopowder oxidation in the ambient air.

**Figure 1:**
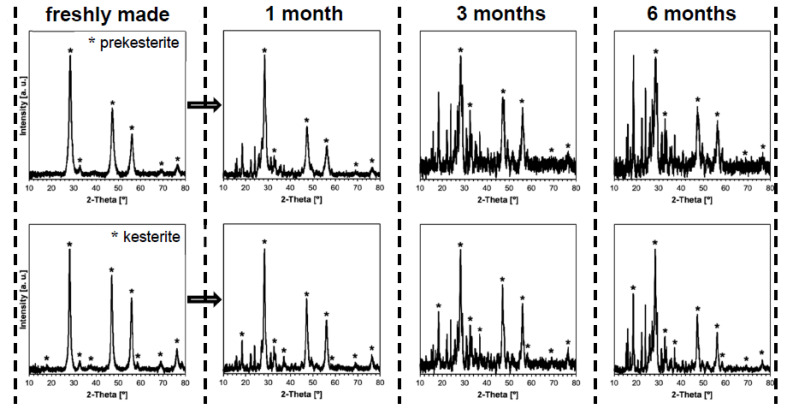
XRD patterns for freshly made and for exposed to ambient air for 1, 3, and 6 months (from left to right, respectively) nanopowders in CE precursor system. The patterns originated from prekesterite are in top row and from kesterite are in bottom row. Asterisks (*) show peaks for the kesterite polytypes while remaining unmarked peaks are for oxidation products.

**Figure 2:**
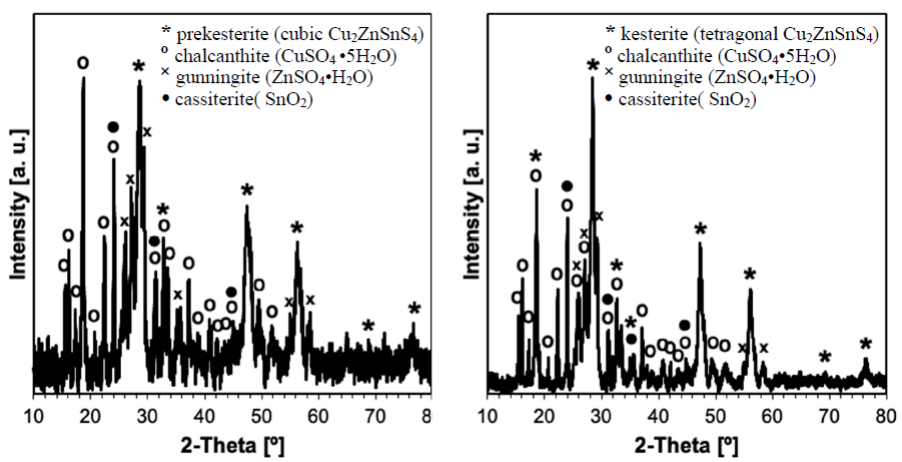
Phase assignments in XRD patterns for 6-month exposed to air nanopowders of prekesterite (**left**) and kesterite (**right**) in CE precursor system. Kesterite polytypes are cubic zincblende-type prekesterite (F43m) and disordered tetragonal kesterite (I42m).

The respective XRD patterns for the nanopowders from the three systems are qualitatively identical when comparing the data from the same stage, i.e., for the freshly prepared powders of prekesterite and kesterite and, in both cases, for their products from the 1, 3 or 6-month air exposure time. The results for the CE precursor system are shown as examples in Figure 1 (all stages) and Figure 2 (6 months, phase assignments) to illustrate the data whereas the structure parameters calculated from all the patterns for all three systems are included in Table 1.

The freshly made nanopowders are the phase pure kesterite polytypes, i.e., cubic (raw) and/or tetragonal (500 °C-annealed) with slightly varying cell parameters that reflect the complex and specific formation chemistry in the different precursor systems. The lower temperature of formation of the raw cubic prekesterite yields an average crystalline size D_av_ in the range 6–9 nm to be compared with the 500 °C-annealed tetragonal kesterites showing on average the doubled D_av_s of 12–18 nm. These data confirm the relatively similar structure parameters within the individual sets of the prekesterite and kesterite and agree well with the relevant parameters reported earlier by us for the products [11,12,13]. During the exposure to ambient air, already after 1 month the progress of oxidation is clearly manifested and the growing with time formation of the hydrated metal sulfates such as CuSO_4_•5H_2_O and ZnSO_4_•H_2_O is confirmed. Interestingly, the only Sn-bearing oxidation product is Sn (IV) oxide SnO_2_ that is convincingly detected only after 6 months. This is consistent with many Sn (IV) compounds to be prone to hydrolysis reactions with the formation of, possibly, hydrated SnO_2_•xH_2_O.

The overall oxidation chemistry in the presence of water vapor can be presented in short in Equation (1).

Cu_2_ZnSnS_4_ + 8O_2_ + 11H_2_O → 2CuSO_4_•5H_2_O + ZnSO_4_•H_2_O + SnO_2_ + SO_2_ (1)

The formation of the most commonly encountered copper sulfate pentahydrate is acknowledged whereas that of the zinc sulfate monohydrate is not as obvious. In the latter case, it is possible that under more humid air conditions or at still longer exposure times other hydrates could form such as the common zinc sulfate heptahydrate. Similarly, tin(IV) oxide could also become hydrated as SnO_2_•xH_2_O. It is interesting to note that the stoichiometry suggests some evolution of toxic gaseous SO_2_ (or SO_3_) during such kesterite oxidation.

In general, the progress of water-assisted oxidation in all precursor systems for the more reactive prekesterite nanopowder is higher than for the related kesterite and after 6 months the remaining amounts of the prekesterites and kesterites relative to the starting materials are in the 31–39 wt% and 44–52 wt% ranges, respectively.

**Figure 3:**
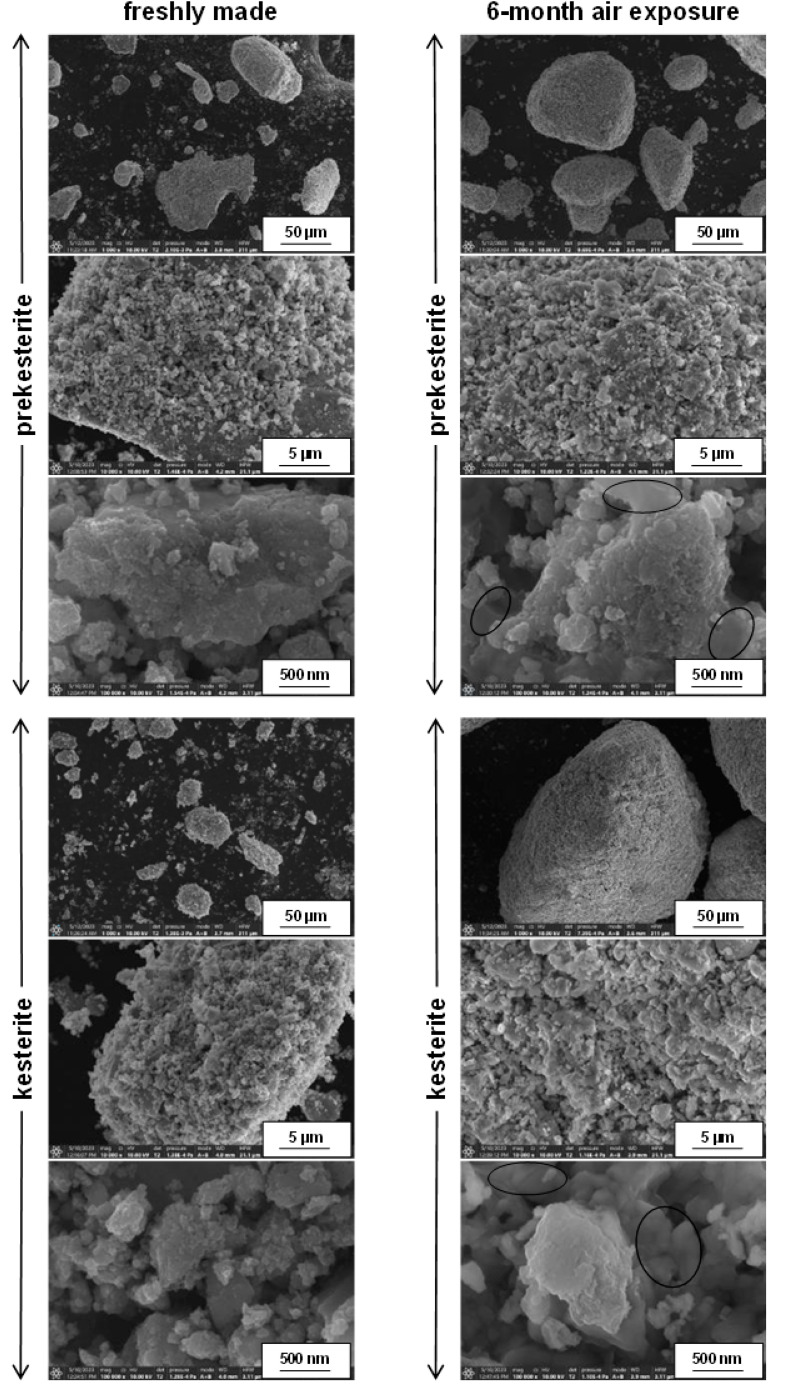
SEM images of freshly made (**left column**) and 6-month exposed to air (**right column**) prekesterite (**top**) and kesterite (**bottom**) nanopowders from MS precursor system. Areas within ovals contain homogeneous/solid features typical for oxidized nanopowders.

The SEM micrographs for the fresh and 6-month exposed to air nanopowders of prekesterite and kesterite from the MS precursor system, which are typical for all systems, are shown in Figure 3. They are selected with a stepwise increase of magnification by a factor of 10 up to the highest magnification in the nanosized range. Both the prekesterite and keterite display similar particle morphology by consisting of a range of submicron to a few micron-large agglomerates that are clearly composed of much smaller objects resolved down to ca. 10 nm in size. The latter are, possibly, single crystallites as supported by the XRD-derived average crystallite sizes (cf. Table 1). Interestingly, the morphology is not much changed after the extensive oxidation of both materials in air. The basic characteristics of the related images are very similar for the fresh and air-exposed nanopowders and there are no clearly observed different morphology features in the latter. A careful examination of the highest magnifications points out, however, to the presence of the quite extensive in the submicron scale homogeneous, solid in appearance regions (see, morphology in the ovals) that are absent in the fresh samples and may correspond to relatively large crystallites of the hydrated metal sulfates. The similar morphology of both kesterite materials suggest that, prevailingly, the oxidation phenomena take place with no drastic impact on the overall particle appearance.

**Figure 4:**
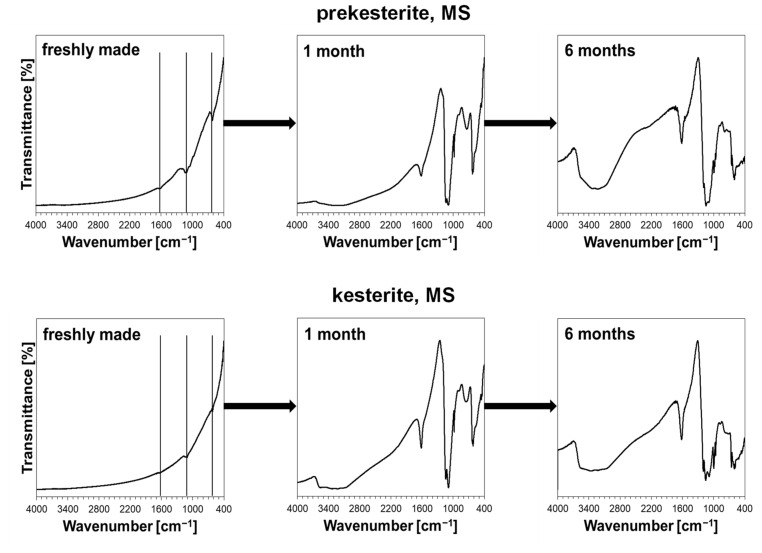
Infrared FT-IR spectra for prekesterite and kesterite nanopowders prepared from MS system as freshly made and as exposed to ambient air for 1 and 6 months. Solid vertical lines are placed for freshly made products in positions of strong bands in hydrated metal (Cu, Zn) sulfates and are guides for eye, only.

The XRD results are corroborated by the FT-IR determinations, all of which are qualitatively similar for the nanopowders from all three systems and a typical case for the MS system is shown in Figure 4. The freshly made nanopowders display very small intensity peaks at ca. 1620, 1100, and 600 cm^−1^, which grow and evolve into more complex bands with exposure time. Additionally, a broad band at 3200–3600 cm^−1^ appears in all spectra after 1 month of exposure. This band and the associated bending mode at 1620–1630 cm^−1^ are typical for the vibrations of O-H in the adsorbed H_2_O molecules or H_2_O molecules of crystallization in the hydrated metal salts. The most intense bands at 1000–1200 and 600–700 cm^−1^ are typical for the hydrated metal sulfates such as CuSO_4_•5H_2_O [42] and ZnSO_4_•H_2_O [43] in agreement with the earlier discussed XRD results. The plausible O-Sn-O symmetric stretching mode in SnO_2_ at ca. 600 cm^−1^ [44] is not easily discernible since it is in the range of the relatively strong vibrations of the sulfate groups. However, the clearly increased with time relative amounts of water are consistent with the formation of SnO_2_ and its propensity to adsorb water vapor forming SnO_2_•xH_2_O. It is evident that the oxidation of both kesterite-type nanopowders is a comparably fast process with the significant effect already after 1-month exposure while further progressing over the 6-month period of time. It is of interest to compare this behavior with the susceptibility to oxidation in ambient air of simple metal sulfides such as Cu_2_S, ZnS, and SnS. In this regard, already after 1-month exposure of the commercial powders of the sulfides there is no FT-IR evidence of the sulfate group formation for ZnS and SnS, whereas strong bands for such groups are found for Cu_2_S. This suggests that the relatively high oxidation reactivity of the copper-sulfur moieties could be a driving force behind similar oxidation of kesterite.

**Figure 5:**
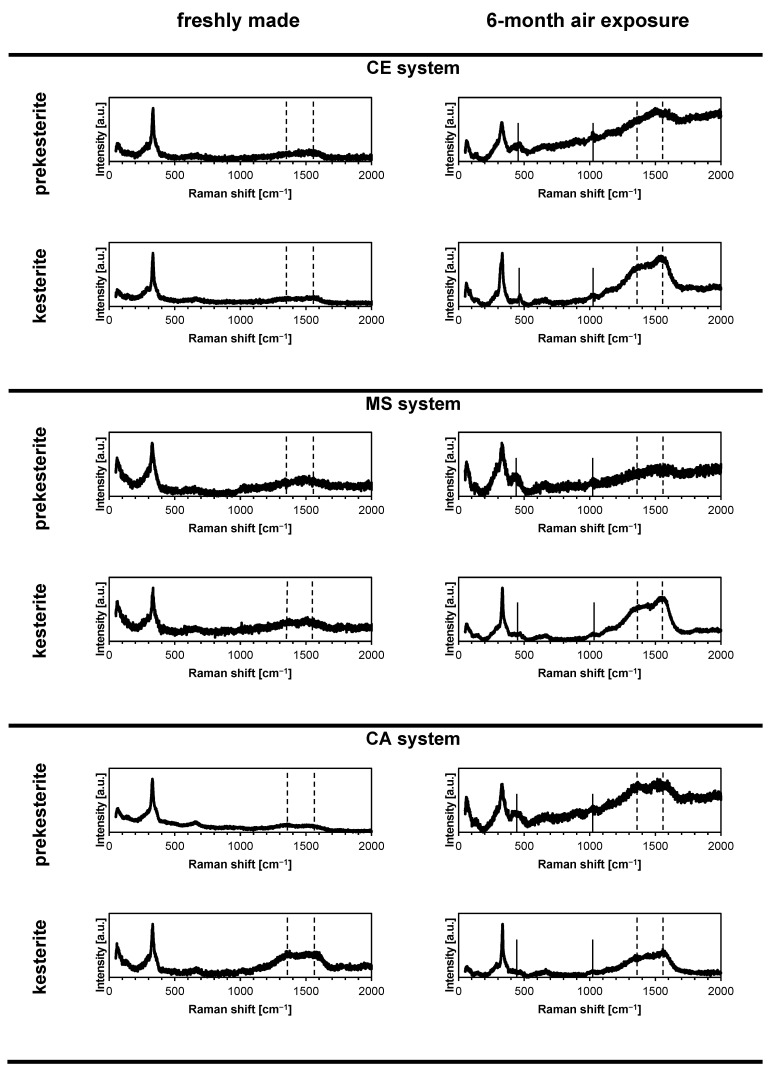
Micro-Raman spectra of the freshly made and 6-month exposed to air nanopowders of prekesterite and kesterite from three precursor systems. Solid vertical lines are in positions of the Raman shifts for sulfate groups and dashed lines are for residual carbon.

The micro-Raman spectroscopy data shown in Figure 5 are also consistent with the partial kesterite oxidation in air and the formation of the copper and zinc sulfates in the nanopowders from all three precursor systems. First, the characteristic Raman bands are similar for the prekesterites and kesterites while for the former they are broader and tend to severely overlap in the diagnostic range 280–360 cm^−1^. The most intense band of A symmetry at 330–338 cm^−1^ is clearly present in all spectra. In particular, the spectra for the prekesterites consist of three peaks at 288–297 cm^−1^ (weak; v. broad), 330–336 cm^−1^ (v. strong; broad), and 350–360 cm^−1^ (v. weak; broad shoulder). The spectra for the kesterites have the respective peaks at 288–293 cm^−1^ (weak; broad), 336–338 cm^−1^ (strong, sharp), and 355–360 cm^−1^ (v. weak; shoulder). There is also frequently seen a weak and broad band at 650–660 cm^−1^ which we tentatively assign as an overtone. Second, in addition to the kesterite related peaks there are two new peaks at 440–470 cm^−1^ (weak, broad) and 1000–1020 cm^−1^ (weak, broad) that are present in the nanopowders exposed to air. These peaks can convincingly be assigned to the presence of the sulfate groups in the hydrated copper and zinc sulfates [45,46] as, specifically, confirmed by XRD and FT-IR. Despite the significant progress of kesterite oxidation after 6 months (cf. Table 1), the remaining quantities of the kesterite polytypes preserve the basic Raman footprint. Third, interestingly, there are also two additional bands seen at ca. 1370 and 1550 cm^−1^ for all materials which are relatively more intense for the annealed kesterite nanopowders. These bands are assigned to the carbon contaminant as, respectively, D and G bands [47], which are reminiscent of the “wet” synthesis conditions with use of the xylene. Apparently, the hydrocarbon is not efficiently evaporated after completion of the mechanochemical synthesis (overnight evaporation in air atmosphere) and its adsorbed remnants undergo cracking reactions in the annealing stage with elemental carbon formation. The presence of the carbon impurity suggests the necessary synthesis modifications such as the evaporation of the “wet” raw product under vacuum using a Schlenk technique (vacuum and inert gas applications).

**Figure 6:**
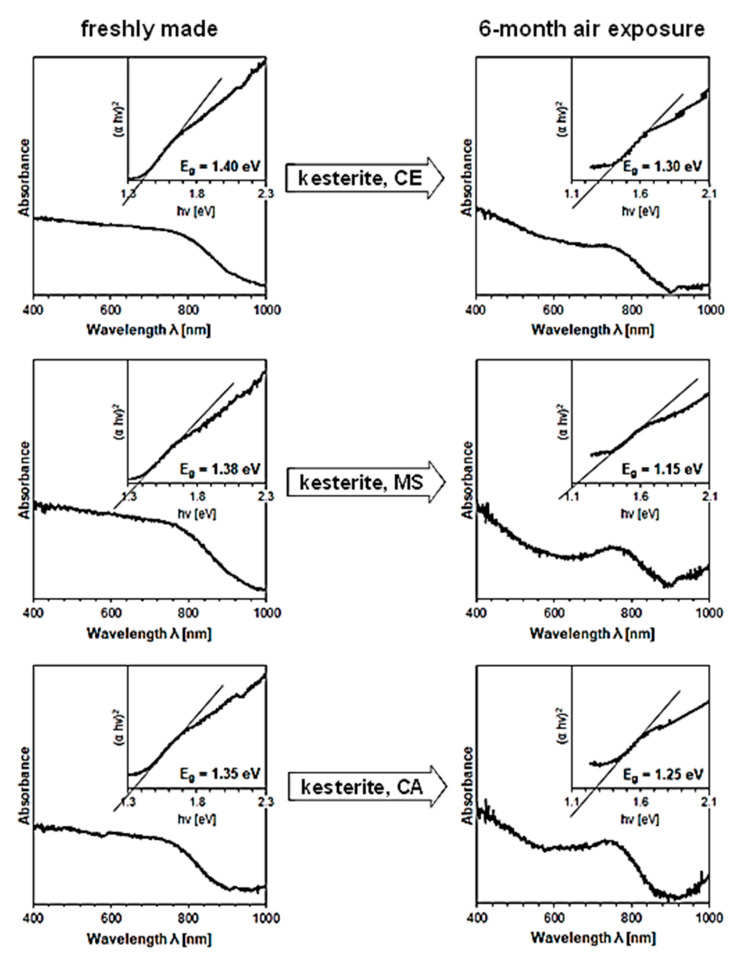
UV-Vis spectra for the freshly made (left column) and exposed for 6 months to ambient air (right column) kesterite nanopowders from the CE (**top**), MS (**middle**), and CA (**bottom**) systems. Spectra have inserts of Tauc (αhν)2 vs. hν [energy] plots (*α* approximated by Kubelka–Munk transformation) and include the calculated energy band gaps Eg.

The UV-Vis spectra were run for all nanopowders and, first, confirmed our earlier observations that the prekesterite nanopowders are defunct of semiconductor properties and yield no specific UV-Vis spectra [11,12,13,14]. Second, the typical spectra were successfully obtained for all three 500 °C-annealed kesterite nanopowders and the specific energy band gaps were calculated from them via Tauc (αhν)2 vs. hν [energy] plots as illustrated in Figure 6 (see, inserts in the spectra) [48]. The energy band gaps show the same trend for all materials, namely, they are clearly lower for those 6-month exposed to air in each pair. Specifically, the decreases of the band gap energy from 1.40 to 1.30 eV for CE system, 1.38 to 1.15 eV for MS system, and 1.35 to 1.25 eV for CA system are observed. It is tempting to assign these changes to the effects of particle oxidation that consumes first the particle surface layers and, therefore, changes the spectroscopic properties of the remaining smaller fragments. Additionally, since the nanopowders naturally have some particle size distribution, the smallest kesterite particles could have been totally oxidized to contribute to changes of the effective spectroscopic output, significantly, towards smaller energy band gaps.

**Figure 7:**
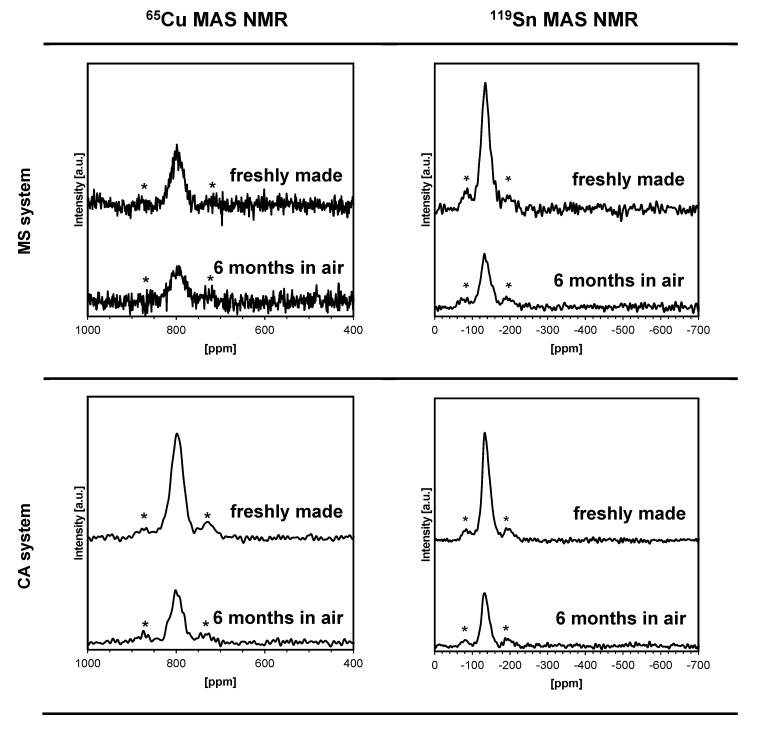
Solid-state ^65^Cu (**left**) and ^119^Sn (**right**) MAS NMR spectra for kesterite nanopowders prepared from the systems MS (**upper part**) and CA (**bottom part**) that were recorded for freshly made and for 6-month exposed to air samples. Asterisks (*) are in positions of spinning side bands.

The solid-state NMR spectroscopy for kesterite has previously provided important yet, admittedly, not well understood information about the material’s defects, intrinsic magnetism, and their impact on semiconducting properties [11,12,13,49]. In this regard, as expected, all prekesterite nanopowders do not produce ^65^Cu/^119^Sn MAS NMR signals which we attribute to intrinsic d_0_ magnetism in the defected fresh particles prepared via the high-energy ball milling. On the other hand, the fresh annealed kesterite nanopowders show the anticipated resonances as exemplified for the systems MS and CA in Figure 7. For both systems, the ^65^Cu and ^119^Sn NMR resonances are determined at the same positions of 799 and −138 ppm, respectively. These spectra are confronted with the spectra collected for the partially oxidized kesterites after 6 months in air to yield for the systems MS and CA, respectively, the ^65^Cu peak positions at 800 and 798 ppm and ^119^Sn peak positions at −134 ppm. For the air exposed nanopowders, it is evident that the resonance intensities fall approximately twice as low compared with the freshly made samples. This is consistent with the XRD estimations for these cases, which support comparable proportions of the remaining kesterite after the air exposure. The important observation is that despite the fact that the oxidation products contain magnetic Cu^+2^ ions in the copper sulfate this does not constitute a sufficiently strong magnetic shielding of the diamagnetic Cu^+1^ centers in the remaining kesterite so to disable resonance conditions. One can infer that the partial oxidation of a kesterite crystallite is mainly a surface phenomenon and does not much effect its core that spectroscopically preserves the characteristic features of the tetragonal kesterite. This is also true with the discussed earlier UV-vis spectra for the exposed to air materials (vide intra).

The helium density and specific surface area data for the fresh and 6-month exposed to air nanopowders are compiled in Table 2. The freshly made prekesterites have somewhat lower densities in the range 3.60–3.90 g/cm^3^ compared to the respective kesterites in the range 3.91–4.30 g/cm^3^ for each of the precursor systems as seen by us previously. This is consistent with the more defected structures of the raw versus annealed kesterite products. The numbers are to be related to the reference density of 4.56 g/cm^3^ for kesterite [50]. A noticeable decrease of the density occurs after exposure to air of both kesterite forms reflecting the prevailing impact of the actual lower densities of the oxidation products such as of dominant quantities of CuSO_4_•5H_2_O (2.29 g/cm^3^) and ZnSO_4_•H_2_O (3.08 g/cm^3^) over the higher density of SnO_2_ (6.95 g/cm^3^) in smaller amounts. Looking from another angle on the morphology features, the BET specific surface areas (calculated from the BET theory) [51], commonly interpreted as total surface areas, for the freshly made prekesterite and kesterite are surprisingly similar to each other being in the range 17.2–18.2 m^2^/g for the CE and MS systems whereas a bit lower values in the range 11.4–12.7 m^2^/g are determined for the CA system. The magnitudes of the BJH specific surface area that is associated with mesopores (calculated from the BJH theory) [52] are similar to the BET data, which is consistent with the notion that all fresh nanopowders are prevailingly mesoporous. The BET/BJH data for the air exposed nanopowders vary systematically in that the areas decrease for the preketerite and increase for the kesterite in each system compared to the respective areas for the fresh materials. Thus, the impact of oxidation on the changes of the specific surface areas is different for each of the kesterite polytypes and appears to be related to the higher oxidation reactivity of the prekesterite and the resultant specific dynamics of the oxidation product formation. Nevertheless, the partially oxidized nanopowders are still mesoporous. In concluding this section, it is obvious that the two morphology-related parameters, i.e., helium density and specific surface areas BET/BJH, are both correlated with the progress of kesterite nanopowder oxidation and they are convenient markers of such changes.

The oxidation of the nanopowders is explicitly evidenced by the direct oxygen and hydrogen determinations as shown in Table 3. First, it is interesting to notice the relatively quite high oxygen O-contents of the order of up to a few wt% in the freshly made nanopowders from all precursor systems, especially, in the raw prekesterites. This is likely to result from some non-strictly standardized manipulation of materials up to the raw product stage. We are inclined to say that the overnight xylene evaporation step could mainly be responsible for it. Given the relatively higher reactivity of the prekesterite, the higher oxidation progress, reflected in the respective higher O-content than in the respective kesterite, is convincingly explained. Additionally, it is worth noting that the increased O-contents are correlated with the higher hydrogen H-contents, which suggests that some of the oxygen is associated with hydrogen, likely, in the H_2_O molecules. Second, most of the oxidation changes take place in the first month of oxidation, whereas at longer times a smaller while still significant oxidation progress is observed. There are abrupt content increases for both oxygen and hydrogen after 1 month in air, respectively, to the levels of 22.1–28.2 and 1.88–2.38 wt%, whereas after the 6-month exposure, relatively smaller content increases are observed. Given the stoichiometry of H_2_O, 1 wt% of H-content is equivalent to 8 wt% of O-content and this can reasonably be used to estimate the proportion of oxygen in the chemisorbed water molecules (e.g., water of crystallization in the sulfates) and in other plausible O-containing moieties (e.g., sulfate groups [SO_4_]^−2^, SnO_2_). From the point of view of kesterite oxidation, all these analytical results are consistent with the earlier discussed experiments.

## 4. Conclusions

The susceptibility to the prolonged oxidation in ambient air of the large pool of kesterite Cu_2_ZnSnS_4_ nanopowders prepared via the mechanochemical synthesis route from three precursor systems was studied by various methods. The major and consistent observation was that already after 1 month of exposure to air a significant progress of a moisture-assisted oxidation took place, which continued with time and, at the end, consumed a half to up to two thirds of the kesterite. The identified oxidation products were the hydrated copper(II) and zinc(II) sulfates, and tin(IV) oxide, also, with the likely release of some toxic gaseous SO_2_. The progress of the changes was relatively faster for the more defected nanopowders of the raw-synthesized cubic polytype compared to the annealed tetragonal kesterite. The remaining post-oxidation kesterite materials still showed the semiconductor properties with a slight shift of up to 0.2 eV of the UV-Vis-determined bandgap to lower levels compared to the freshly made semiconductor. Facing the kesterite nanopowders quite high oxidation propensity in ambient air, the proper attention should be given to suitably standardized and strictly controlled oxygen/water vapor-content conditions during the synthesis, characterization, manipulation, and storage of this nanosized material.

## Figures and Tables

**Table 1:** Lattice constants *a* and *c*, average crystallite sizes *D_av_*, and wt% proportions of kesterite phases (cubic prekesterite and tetragonal 500 °C-annealed kesterite) in fresh and oxidized nanopowders prepared from three precursor systems. The data are for freshly made and for 1, 3, and 6-month exposed to air nanopowders.

	Cubic Zincblende-Type Prekesterite			Disordered Tetragonal Kesterite		
	a [Å]	D_av_ [nm]	wt%	a/c [Å]	D_av_ [nm]	wt%
**CE system**						
freshly made	5.44	6	100	5.43/10.86	12	100
1 month	5.42	12	70	5.43/10.96	12	66
3 month	5.45	9	42	5.45/10.98	12	46
6 month	5.42	11	39	5.42/10.88	13	44
**MS system**						
freshly made	5.42	7	100	5.43/10.83	13	100
1 month	5.43	9	62	5.42/10.94	13	65
3 month	5.44	12	43	5.44/10.98	12	57
6 month	5.41	9	38	5.44/10.93	12	50
**CA system**						
freshly made	5.43	9	100	5.44/10.82	18	100
1 month	5.42	9	55	5.42/10.90	14	66
3 month	5.44	9	36	5.44/10.99	15	65
6 month	5.41	11	31	5.43/10.93	14	52

**Table 2:** Helium density and BET/BJH specific surface area data for freshly made and 6-month air exposed kesterite-type nanopowders from three precursor systems. Note that BET (Brunauer-Emmett-Teller) corresponds to the total surface area and BJH (Barrett-Joyner-Halenda) to the mesopore area.

	CE System		MS System		CA System	
	Prekesterite Kesterite		Prekesterite Kesterite		Prekesterite Kesterite	
	HELIUM DENSITY d_He_ [g/cm^3^]					
freshly made	3.64	3.91	3.90	4.24	3.6	4.3
6-month exposure	3.12	3.25	2.86	3.25	3.04	3.51
	BET/BJH SPECIFIC SURFACE AREA [m^2^/g]					
freshly made	18.2/21.9	17.3/21.1	17.9/21.9	17.2/21.1	12.7/13.0	11.4/11.6
6-month exposure	12.0/12.8	21.8/25.9	10.6/11.7	21.1/24.2	11.4/11.8	19.5/21.9

**Table 3:** Directly determined oxygen and hydrogen contents in the freshly made and 1, 3, and 6-month exposed to air kesterite nanopowders from three precursor systems.

	CE System		MS System		CA System	
	Prekesterite	Kesterite	Prekesterite	Kesterite	Prekesterite	Kesterite
OXYGEN CONTENT [wt%]						
freshly made	4.48	0.63	5.86	1.16	4.32	1.60
1 month	22.4	25.4	22.1	24.7	28.2	24.8
3 month	28.7	31.4	31.2	33.3	32.2	33.4
6 month	31.8	36.3	33.3	32.8	33.6	34.0
HYDROGEN CONTENT [wt%]						
freshly made	0.30	0.01	0.32	0.02	0.46	0.04
1 month	2.04	2.26	1.88	2.29	2.38	2.20
3 month	2.38	2.44	2.38	2.37	2.58	2.30
6 month	3.00	2.79	3.06	2.78	3.04	2.67

## Data Availability

Data available on request from the corresponding author.
